# Potential correlations between abnormal homogeneity of default mode network and personality or lipid level in major depressive disorder

**DOI:** 10.1002/brb3.3622

**Published:** 2024-07-17

**Authors:** Chunguo Zhang, Feichao Ruan, Haohao Yan, Jiaquan Liang, Xiaoling Li, Wenting Liang, Yangpan Ou, Caixia Xu, Guojun Xie, Wenbin Guo

**Affiliations:** ^1^ Department of Psychiatry The Third People's Hospital of Foshan Foshan Guangdong China; ^2^ Department of Psychiatry National Clinical Research Center for Mental Disorders and National Center for Mental Disorders The Second Xiangya Hospital of Central South University Changsha Hunan China

**Keywords:** default mode network, magnetic resonance imaging, major depressive disorder, network homogeneity, personality

## Abstract

**Background:**

Default mode network (DMN) is one of the most recognized resting‐state networks in major depressive disorder (MDD). However, the homogeneity of this network in MDD remains incompletely explored. Therefore, this study aims to determine whether there is abnormal network homogeneity (NH) of the DMN in MDD patients. At the same time, correlations between clinical variables and brain functional connectivity are examined.

**Methods:**

We enrolled 42 patients diagnosed with MDD and 42 HCs. A variety of clinical variables were collected, and data analysis was conducted using the NH and independent component analysis methods.

**Results:**

The study shows that MDD patients have higher NH values in the left superior medial prefrontal cortex (MPFC) and left posterior cingulate cortex (PCC) compared to HCs. Additionally, there is a positive correlation between NH values of the left superior MPFC and Eysenck Personality Questionnaire values. NH values of the left PCC are positively linked to CHOL levels, LDL levels, and utilization scores. However, these correlations lose significance after the Bonferroni correction.

**Conclusion:**

Our findings indicate the presence of abnormal DMN homogeneity in MDD, underscoring the significance of DMN in the pathophysiology of MDD. Simultaneously, the study provides preliminary evidence for the correlation between clinical variables and brain functional connectivity.

## 1 INTRODUCTION

Depression poses a considerable global mental health challenge and stands as a key contributor to mental health‐related disabilities on a worldwide scale (Herrman et al., 2019). The lifetime prevalence of major depressive disorder (MDD) reaches up to 20% (Kessler et al., 2005). Many individuals experiencing depression often encounter its initial onset during the later stages of adolescence, typically spanning from 14 to 25 years old. Within this age range, the median 12‐month prevalence varies between 4% and 5% (Pinquart & Duberstein, 2010). MDD has adverse effects on academic endeavors, interpersonal relationships, and occupational engagements. Furthermore, it is prospectively associated with various health conditions, encompassing obesity, cardiac diseases, and premature mortality, including suicide (Hasler et al., 2005; Nicholson et al., 2006). Among individuals aged 18 and above, the impact of MDD on daily functioning can be notably severe. In this demographic, the coexistence of depression with physical health issues not only amplifies its effects but also adds complexity when evaluating treatment options (Marwaha et al., 2023). Despite the high prevalence and substantial impact of MDD, the neurophysiology of MDD remains an ongoing area of exploration.

In the past two decades, neuroimaging methodologies, particularly functional magnetic resonance imaging (fMRI), have been employed to unravel the fundamental neurophysiological aspects of MDD. Recent research findings have solidified the connection between MDD and the dysregulation of network functions, specifically within the cognitive control network (Shou et al., 2017), dorsal attention network (Gao et al., 2023), sensory‐motor networks (Zhang et al., 2023), and default mode network (DMN) (Cui et al., 2021). Among these networks, the DMN is highlighted as playing a pivotal role in the neurophysiology of MDD (Cui et al., 2017; Guo et al., 2013).

Comprising diverse brain regions, the DMN includes the precuneus/posterior cingulate cortex (PCC), medial prefrontal cortex (MPFC), and the medial, lateral, and inferior parietal lobules (Raichle et al., 2001). The DMN has been a focal point in numerous studies. However, inconsistencies arise in the findings, with certain studies emphasizing increased resting‐state functional connectivity (rsFC) within the DMN (Greicius et al., 2007; Hamilton et al., 2011; Zhou, Wang et al., 2010), whereas others indicate a reduction in rsFC within the DMN (Anand et al., 2005; Veer et al., 2010; Yan et al., 2019). Moreover, the observed functional changes extend beyond MDD patients, manifesting across diverse age groups (Andreescu et al., 2013; Gaffrey et al., 2012), various stages of the illness (Chen et al., 2015), and in individuals at high risk for depression (Posner et al., 2016). The review highlights the pivotal role of network dysfunction or disruption in the neurobiological mechanisms of severe depression. Substantial support in relevant research affirms the core functions of the DMN and the interruption of its functional connections with multiple brain regions (Brakowski et al., 2017). Furthermore, the neurophysiology of the DMN in MDD may also be affected, such as the combined alterations in ALFF of the left insula and regional cerebral blood flow may represent core neuropathological changes in medication‐free MDD patients (Li et al., 2017). Furthermore, cortical thickness of the orbitofrontal cortex in MDD patients may decrease (Myung et al., 2016), or reduced subcortical white matter connections in the frontal lobe of severe MDD patients may be associated with suicidal ideation (Schmaal et al., 2017). Given the complexity of MDD, current research has not completely clarified its underlying mechanisms, necessitating further extensive exploration.

Network homogeneity (NH), a method proposed by Uddin et al. (2008) for analyzing functional neuroimaging data, represents an informative approach. It provides an unbiased exploration of a distributed network of interest and identifies brain regions indicative of disease‐related disruptions in network coherence. Unlike traditional FC analysis, NH offers an impartial assessment of NH without the need for prior knowledge of the network's location. NH measures the correlation between a given voxel and others in a local or whole‐brain network of interest at the voxel level. In other words, this method primarily investigates functional connectivity between specific voxels within a particular network such as the DMN (Li, Teng et al., 2021). This approach also presents a potential alternative for evaluating the homogeneity of specific large‐scale networks (Guo et al., 2014; Uddin et al., 2008). Studies propose that under specific conditions, individuals exhibit variations in the temporal homogeneity of voxels within distinct functional brain networks. A decline in NH may signal potential instability in the brain network (Yan et al., 2022). Hence, analyses of NH can be employed to assess abnormal alterations in neurological activity within a designated brain network (Gao et al., 2023).

Certain studies have illustrated that particular demographic and clinical factors correlate with cognitive impairment in MDD. These factors include the onset and duration of the disorder, the number of hospitalizations, depression severity, and the subtype of depression (Elderkin‐Thompson et al., 2006; Hasselbalch et al., 2012; Nandrino et al., 2002), as well as age (Hasselbalch et al., 2011) and education (McLaren et al., 2015). A review proposes that examining individual and clinical variables could offer a dependable approach for the personalized selection of antidepressant medications tailored to individuals experiencing severe depression (Perna et al., 2020). Studying the correlation between abnormal DMN homogeneity and clinical variables may represent a new direction in exploring MDD.

This study employed the NH method to investigate the DMN homogeneity in patients with MDD and HCs. We speculate that there may be anomalies in DMN homogeneity in the MDD group, whereas such anomalies may not be present in the control group. Furthermore, we hypothesize that the aberrant DMN homogeneity is potentially correlated with clinical variables in individuals with MDD.

## 2 METHODS AND MATERIALS

### 2.1 Subject

This study was approved by the Clinical Research Ethics Committee of the Third People's Hospital of Foshan, and written informed consent was obtained from all participants (Ethics approval number: FSSY‐LS202106).

Depressive participants in this study were enlisted from the Third People's Hospital of Foshan, including 36 first‐episode and 10 recurrent depressive individuals. The diagnosis of MDD was established using the patient version of the Diagnostic and Statistical Manual of Mental Disorders‐5 (DSM‐5) patient version (American Psychiatric Association, 2013). Recurrent patients voluntarily refrained from antidepressant use for a minimum of 2 weeks. Concurrently, 44 healthy controls (HCs), matched for gender and years of education, were recruited from the community.

All subjects underwent assessments using the Hamilton Anxiety Scale (Bruss et al., 1994), Hamilton Depression Scale (Bagby et al., 2004), Social Disability Screening Schedule (Huang et al., 2022), Eysenck Personality Questionnaire (EPQ) (Smith & Ellingson, 2002), Simplified Coping Style Questionnaire (Cai et al., 2021), Social Support Scale (Li & Shou, 2021), Wisconsin Card Sorting Test (Miles et al., 2021), and Repeatable Battery for the Assessment of Neuropsychological Status (Faust et al., 2017) to measure anxiety and depressive symptoms, social function, personality characteristics, coping style, social support, and psychological cognitive function.

All participants were right‐handed. Exclusion criteria for all subjects were as follows: (1) severe physical illness or disability, (2) concurrent severe psychiatric disorders such as schizophrenia or schizoaffective disorder, and (3) history of alcohol or drug abuse.

### 2.2 Event‐related potentials (ERP) and exploratory eye movement (EEM) data acquisition

Event‐related potential data were collected using a Kohden MEB‐9402C myoelectric evoked potentiometer. Participants were instructed to sit comfortably and concentrate. Electrode placement followed the 10/20 standard, with the ground at FPz and recording electrode at the right ear M2 point. Reference electrode was at the central Cz point. Impedance was set to 5 K, and the filter ranged from 0.2 to 20 Hz. Stimulation was in “Oddball” auditory mode at 1 Hz, 10 ms duration, and 5 µV sensitivity. Nontarget stimuli had 80% probability, 70 dB intensity, and 1000 Hz frequency, whereas target stimuli had 20% probability, 90 dB intensity, and 2000 Hz frequency. Latencies of N100, P200, N200, and P300 waves were recorded separately. For details, please refer to our previously published research (Zhang et al., 2023).

Exploratory eye movement data were collected using a Dekang DEM‐2000 eye movement detector. Participants sat comfortably and focused on a screen displaying S‐shaped patterns. Gaze positions and eye fixations (NEF) were automatically recorded during a 15‐s display of each pattern. Participants then identified differences between subsequent patterns (S2 and S3) and responded with “There is no difference.” The responsive seeking score (RSS) was calculated from gaze points in seven locations over 5 s. An NEF of 30 and/or RSS of 4 indicated abnormality. The device recorded eye movement trajectories, and data were automatically processed for later analysis. For details, please refer to our previously published research (Zhang et al., 2023).

### 2.3 Detection of blood

Using the Shenzhen fully automatic biochemical analysis instrument Merit CL6000i, blood lipids are evaluated according to standardized procedures. First, 5 mL of peripheral blood samples are collected from each participant and allowed to coagulate. Subsequently, serum is separated from blood cells by centrifugation and transferred to clean, sterile tubes. Analysis reagents are then prepared according to the manufacturer's guidelines. The Merit CL6000i employs an endpoint method, catalyzing reactions between cholesterol esterase, cholesterol oxidase, peroxidase, and cholesterol in the sample, producing hydrogen peroxide. This compound then reacts with a chromogenic substrate, generating colored compounds quantified by the CL6000i. Finally, the analyzer outputs all test data.

### 2.4 Imaging data acquisition

MRI data were obtained using a GE 3.0 T scanner (GE 3.0 T Signa Pioneer). Participants were instructed to remain still, close their eyes, and stay alert. Soft earplugs and foam pads were employed to minimize the impact of scanner noise and head motion during imaging. Imaging parameters included a repetition time/echo time of 2000/30 ms, 36 slices, a 64 × 64 matrix, a flip angle of 90°, a 24 cm field of view, a 4 mm slice thickness with no gap, and a total of 250 volumes (500 s).

### 2.5 Data preprocessing

We preprocessed the data using Matlab (Mathworks) and the Data Processing Assistant for Resting‐State fMRI (DPARSF). After correcting for slice timing and head motion, analyzed subjects exhibited a maximum displacement of ≤2 mm in any direction and an angular rotation of <2° on each axis. Scans underwent normalization to the standard SPM12 echo‐planar imaging template through both linear and nonlinear registration steps. Linear registration aligned the entire brain to a template using a 12‐parameter affine transformation, whereas nonlinear registration employed a Bayesian framework for maximum correctness probability. Nonlinear deformations were estimated by combining three‐dimensional discrete cosine transform basis functions, representing deformation coefficients in three directions. The matching process minimized bending energies and residual squared differences. Following spatial normalization, images were resampled to 3 mm × 3 mm × 3 mm dimensions, and temporal bandpass filtering (0.01–0.08 Hz) and linear detrending were applied. Linear regression removed spurious covariates, including 24 head motion parameters and signals from ventricular and white matter regions, along with their temporal derivatives. The ongoing debate on including or excluding the global signal in preprocessed resting‐state FC data is acknowledged, with this study opting to retain the global signal (Guo et al., 2014).

### 2.6 DMN identification

The group independent component analysis (ICA) method was utilized to identify the DMN as a mask from HC subjects. This involved three primary steps: data reduction, independent component (IC) separation, and back reconstruction, performed using the GIFT toolbox (http://mialab.mrn.org/software/#gica). To start, we conducted subject‐ and group‐level principal component analyses (PCAs) to reduce dimensions. The number of ICs was determined using the minimum description length criterion (Li et al., 2007), set at 20 for this study. Next, we employed a back‐reconstruction strategy to obtain specific ICs from the group ICs and PCA reduction outcomes (Erhardt et al., 2011). For all participants, two DMN components were chosen based on GIFT templates (Raichle, 2015). Subsequently, statistical maps were thresholded using voxel‐wise one‐sample *t*‐tests for each component (*p* < .05, corrected for multiple comparisons via Gaussian random field [GRF] theory, with voxel significance at *p* < .001 and cluster significance at *p* < .05) (Song et al., 2011). Two masks were generated and then combined to create a unified DMN mask, employed in subsequent NH analyses.

### 2.7 NH analyses

NH analyses were conducted in Matlab (Mathworks). Voxel‐wise NH was calculated for each participant based on the equation outlined in a previous study (Uddin et al., 2008). The mean NH for each voxel within the DMN mask was identified, and the resultant NH maps underwent smoothing with a Gaussian kernel having a full‐width at half‐maximum of 8 mm.

### 2.8 Statistical analyses

This study employed SPSS version 25.0 for data analysis. Gender differences between groups were assessed using the chi‐square test. Continuous variables, including age, years of education, and clinical scales, underwent comparison through two‐sample *t*‐tests. The significance level was established at *p* < .05.

Following confirmation of NH data normality (*p* > .5) using REST software, NH analyses were conducted. Two‐sample *t*‐tests were employed, utilizing voxel‐wise cross‐subject statistics within the DMN. The significance level was established at a corrected *p* < .05 for multiple comparisons based on GRF theory (voxel significance: *p* < .001, cluster significance: *p* < .05). Acknowledging the potential impact of small movements on resting‐state FC, framewise displacement (FD) values were computed for each subject. In the group comparison of NH, mean FD, age, sex, and educational level served as covariates.

## 3 RESULTS

### 3.1 Demographic and clinical data

**FIGURE 1:**
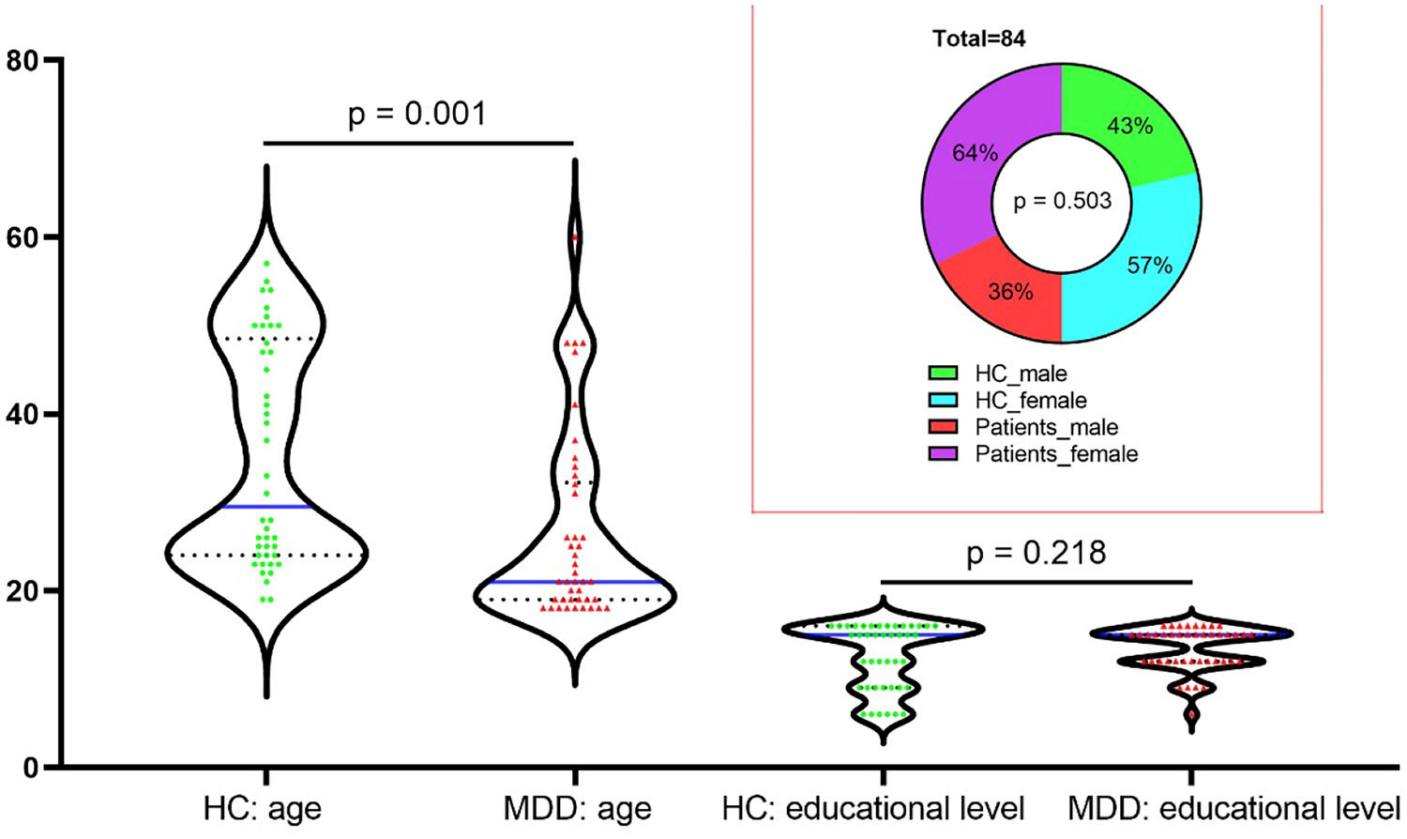
The age, gender, and educational level of patients with major depressive disorder and healthy controls.

This study initially included 46 patients with MDD and 44 HCs. However, due to considerable head movement, data from two HCs and four patients were excluded. Consequently, the final imaging analysis comprised 42 MDD patients and 42 HCs. Detailed demographic and medical information for the individuals can be found in Table 1 and Figure 1. Notably, there was a significant age difference between patients and HCs (*p* = .01), whereas no significant differences were observed in sex and years of education.

**TABLE 1:** Characteristics of participants.

Variables	Patients (*n* = 42)	Controls (*n* = 42)	*p*‐Value
Age (years)	26.43 ± 10.79	35.14 ± 12.54	.001^a^
Sex (male/female)	15/27	18/24	.503^b^
Years of education (years)	13.48 ± 2.48	12.62 ± 3.72	.218^a^
HAMD	24.80 ± 7.22	2.55 ± 3.54	<.001^a^
HAMA	16.60 ± 5.70	2.03 ± 2.69	<.001^a^
EPQ			
P	51.20 ± 8.19	47.23 ± 12.68	.092^a^
E	40.16 ± 11.42	48.72 ± 13.93	.003^a^
N	68.37 ± 9.19	45.08 ± 10.01	<.001^a^
L	44.92 ± 11.30	56.93 ± 11.62	<.001^a^
SDSS total score	7.07 ± 2.42	0.02 ± 0.15	<.001^a^
SSS			
Total score	28.60 ± 8.71	43.14 ± 9.33	<.001^a^
Objective support score	7.45 ± 3.41	10.93 ± 2.85	<.001^a^
Subjective support score	14.50 ± 5.23	23.36 ± 5.91	<.001^a^
Utilization of support	6.64 ± 2.12	8.86 ± 2.05	<.001^a^
SCSQ			
Total score	26.83 ± 8.42	29.90 ± 9.43	.119^a^
Active coping	16.50 ± 6.19	22.90 ± 7.35	<.001^a^
Negative coping	10.33 ± 4.24	7.00 ± 4.35	.001^a^
WCST			
CC	5.00 ± 1.21	5.26 ± 1.23	.328^a^
RA	46.05 ± 2.71	44.00 ± 4.02	.008^a^
RC	34.55 ± 5.58	34.93 ± 3.58	.711^a^
RE	11.50 ± 6.88	9.02 ± 6.21	.087^a^
RP	3.57 ± 4.94	2.14 ± 3.33	.124^a^
RPE	1.79 ± 2.85	0.81 ± 1.45	.052^a^
RBANS			
Immediate memory	42.15 ± 10.40	42.83 ± 11.69	.781^a^
Visuospatial/constructional	18.73 ± 2.16	17.90 ± 2.29	.100^a^
language	17.60 ± 4.43	18.57 ± 4.20	.311^a^
Attention	60.60 ± 14.11	64.81 ± 16.09	.212^a^
Delayed memory	48.33 ± 9.65	49.60 ± 10.66	.574^a^
EEM			
NEF	21.11 ± 5.95	27.52 ± 4.32	<.001^a^
RSS	3.71 ± 1.51	4.60 ± 1.62	.025^a^
ERP			
N100	103.73 ± 15.35	107.86 ± 34.86	.508^a^
P200	174.53 ± 21.59	177.62 ± 23.66	.545^a^
N200	230.21 ± 31.52	211.79 ± 44.69	.038^a^
P300(ms)	309.74 ± 24.20	287.10 ± 50.80	.012^a^
TG (mmol/L)	0.975 ± 0.65	1.463 ± 2.51	.226^a^
CHOL (mmol/L)	4.377 ± 1.05	4.651 ± 0.72	.163^a^
HDL (mmol/L)	1.323 ± 0.39	1.248 ± 0.30	.333^a^
LDL (mmol/L)	2.410 ± 0.84	2.615 ± 0.58	.199^a^
FBG (mmol/L)	5.521 ± 1.95	5.639 ± 0.94	.729^a^
TSH (mIU/L)	1.650 ± 0.83	2.171 ± 0.90	.007^a^
FT3 (pmol/L)	4.380 ± 0.80	4.801 ± 0.57	.006^a^
FT4(pmol/L)	16.950 ± 14.64	14.551 ± 2.98	.301^a^

Abbreviations: CC, categories completed; CHOL, total cholesterol; E, extraversion; EEM, exploratory eye movement; EPQ, Eysenck Personality Questionnaire; ERP, event related potential; FBG, fasting blood glucose; FT3, free triiodothyronine; FT4, free thyroxine; HAMA, Hamilton Anxiety Rating Scale; HAMD, Hamilton Depression Rating Scale; HDL, high‐density lipoprotein; L, Lie; LDL, low‐density lipoprotein; N, neuroticism; NEF, number of eye fixation; P, psychoticism; RA, responses answer; RBANS, Repeatable Battery for the Assessment of Neuropsychological Status; RC, correct responses; RE, errors responses; RP, perseverative responses; RPE, perseverative responses errors; RSS, responsive search score; SCSQ, Simplified Coping Style Questionnaire; SDSS, social disability screening schedule; SSS, Social Support Revalued Scale; TG, triglycerides; TSH, thyroid‐stimulating hormone; WCST, Wisconsin Card Sorting Test.

^a^ The *p*‐values were obtained by two samples *t*‐tests.

^b^ The *p*‐value for sex distribution was obtained by a chi‐square test.

### 3.2 DMN maps determined by ICA

**FIGURE 2:**
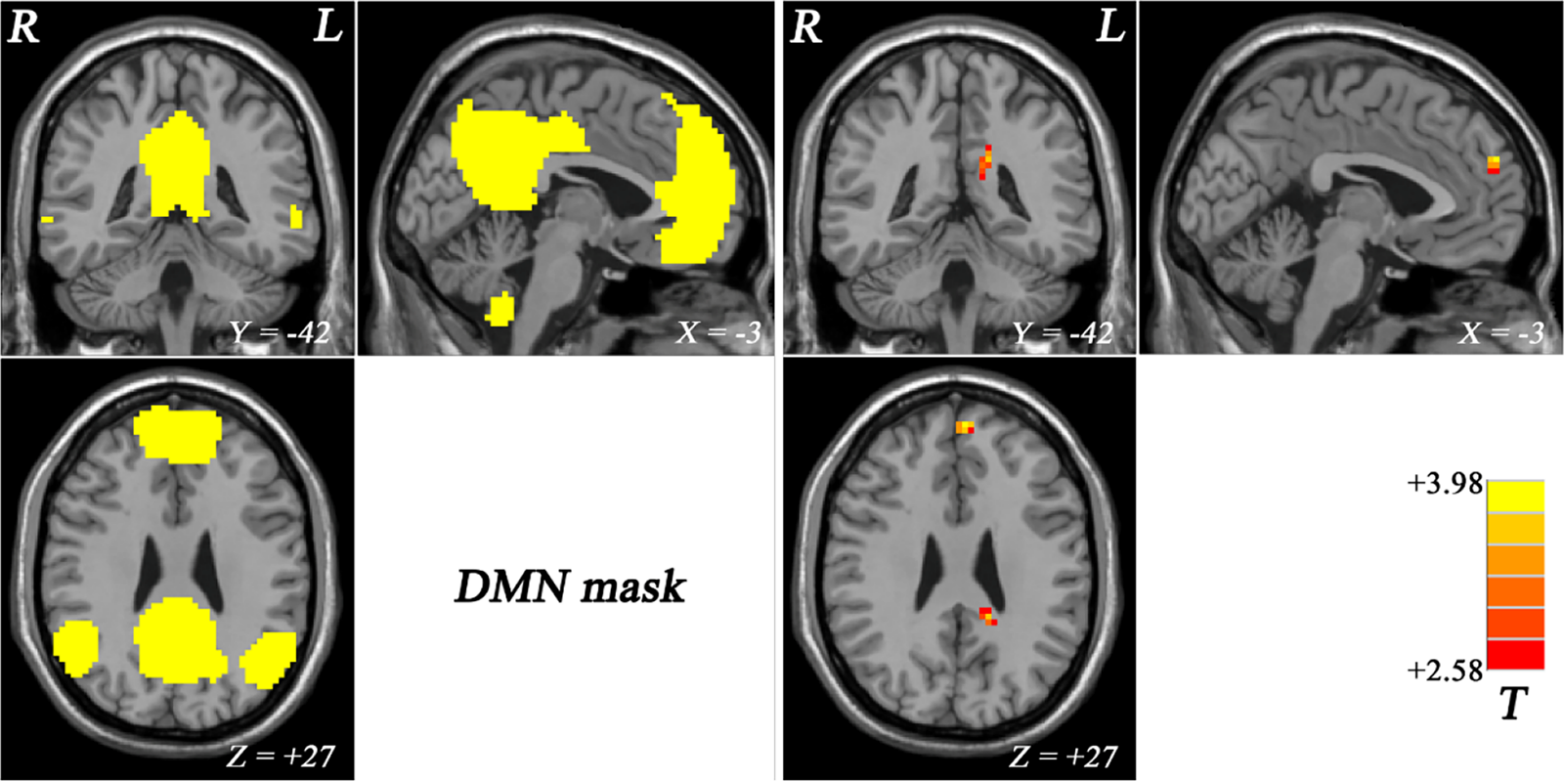
The DMN mask determined by ICA from the control group and statistical maps showing NH differences between subject groups (major depressive disorder [MDD] > HC). Red denotes higher NH. Colored bars indicate the *T* value from two‐sample *t*‐tests. DMN ,  default mode network; ICA,  independent component analysis; NH ,  network homogeneity.

A group ICA approach was employed to select the DMN from the control subjects. The identified DMN was subsequently utilized as a mask in the subsequent NH analyses, encompassing specific brain regions (Figure 2).

### 3.3 NH: group differences in the DMN

Two‐sample *t*‐tests, engaging voxel‐wise cross‐subject comparisons, revealed a significant group difference in NH values within the DMN between patient and control subjects. Specifically, NH values in the left superior MPFC and left PCC were higher in patients compared to HCs (Figure 2 and Table 2).

**TABLE 2:** Brain regions with increased DMN homogeneity in the patients.

Cluster location	Peak (MNI)			Number of voxels	*T* value
	*x*	*y*	*z*		
Left superior MPFC	−3	57	27	38	3.9759
Left PCC	−15	−42	27	30	3.6178

Abbreviations: DMN, default mode network; MNI, montreal neurological institute; MPFC, medial prefrontal cortex; PCC, posterior cingulate cortex.

### 3.4 Correlations between NH and clinical variables

**FIGURE 3:**
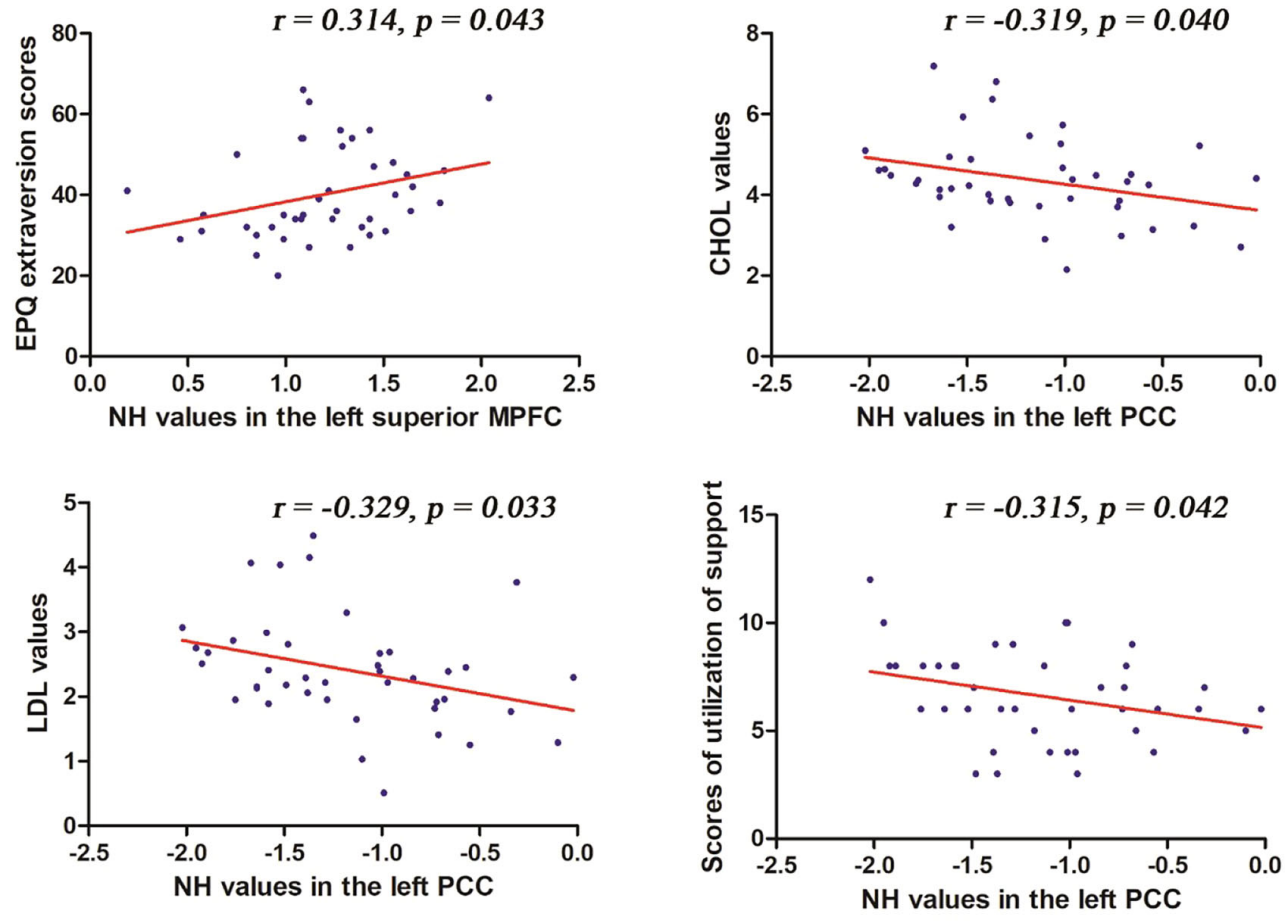
Correlations between network homogeneity (NH) and clinical variables. CHOL, total cholesterol; EPQ, Eysenck Personality Questionnaire; LDL, low‐density lipoprotein; MPFC, medial prefrontal cortex; PCC, posterior cingulate cortex.

The study results indicate a positive correlation between NH values of the left superior MPFC and EPQ_E values (*r* = .314, *p* = .043). Additionally, NH values of the left PCC show positive correlations with CHOL (cholesterol) levels (*r* = −.314, *p* = .040), LDL (low‐density lipoprotein) levels (*r* = −.329, *p* = .033), and scores of utilizations of social support (*r* = −.315, *p* = .042) (Figure 3). However, these correlations lost significance after the Bonferroni correction. Meanwhile, we conducted correlation analyses for the control group with the same brain regions and clinical variables. The left superior MPFC NH value showed no correlation with EPQ_E value (*r* = .188, *p* = .234), and the left PCC NH value exhibited no correlations with CHOL levels (*r* = .138, *p* = .388), LDL levels (*r* = .1124, *p* = .485), and scores of utilization of support (*r* = .030, *p* = .849).

In addition, we have tested the influence of age on brain‐behavior correlations using partial correlation with NH values and clinical assessments adjusted for age. We found that only the correlation between NH values of the left superior MPFC and EPQ_E values (*r* = .313, *p* = .047) remained significant after adjustment for age (Figure S1). However, the other three correlations were no longer significant: the correlation between NH values of the left PCC and CHOL (cholesterol) levels (*r* = −.254, *p* = .108), the correlation between NH values of the left PCC and LDL levels (*r* = −.232, *p* = .144), and the correlation between NH values of the left PCC and scores of utilization of social support (*r* = −.277, *p* = .079).

Finally, we performed a PCA to reduce the dimensionality of 39 clinical variables listed in Table 1 (excluding age, gender, and educational level). Eleven factors were identified, explaining a total of 88.96% of the variance. However, no factor correlated with the NH values in the left superior MPFC or left PCC. Furthermore, after controlling for age, no correlation was found between these 11 factors and the NH values in the left superior MPFC or left PCC. Please see the Figure S2 and Tables S1 and S2 for the results.

## 4 DISCUSSION

Our study demonstrates that, compared to HCs, patients with MDD exhibit higher NH values in the DMN. Simultaneously, the study indicates a positive correlation between NH values of the left superior MPFC and EPQ values. NH values of the left PCC are positively associated with CHOL levels, LDL levels, and scores of utilizations of social support. However, it is noteworthy that these correlations lose significance after applying the Bonferroni correction.

Our prior investigations have shown that the DMN's FC in individuals with MDD exhibits alterations (Guo et al., 2018; Yan et al., 2022, 2021). Specifically, fMRI studies reveal a distinctive neural network pattern in individuals experiencing gastrointestinal symptoms within the depressed patient population (Yan et al., 2022). Concurrently, research suggests that individuals with depression manifest heightened activity in the DMN during self‐referential thinking processes (Sheline et al., 2009). Importantly, this elevated activity persists even when engaged in tasks demanding attention (Anticevic et al., 2012). Recent findings propose disrupted dynamics within the DMN during emotional processing in MDD. This emphasizes a potential pathology linked to emotion within the DMN, potentially tied to rigidly sustained emotional responses. Recognizing this association holds significant importance in unraveling the risk factors associated with depression (Provenzano et al., 2021). Furthermore, the therapeutic impact of antidepressants includes a reduction in excessive connectivity within the DMN among MDD patients, aligning it with levels observed in HCs (Posner et al., 2013). This indicates that the connectivity within the DMN plays a crucial role in patients with MDD. Furthermore, based on a previous replication study, we found increased NH in the DMN overlap region among first‐episode, drug‐naive MDD patients experiencing their first episode across two independent samples (Guo et al., 2018).

The MPFC, in conjunction with the PCC, constitutes the central hub of the DMN (Raichle et al., 2001). Specifically, the MPFC plays a pivotal role in social cognition and the processing of emotions (Etkin et al., 2011). The MPFC has been shown to play a significant role in modulating emotional behavior and self‐referential processing in MDD, as evidenced by both task‐related and resting‐state functional neuroimaging methods (Drevets et al., 2008; Sheline et al., 2009). The superior MPFC serves as a crucial node within both the DMN and the dorsal cognitive system. Elevated NH in the left superior MPFC may impact the functionality of this region (Northoff, 2007). Our previous research also confirmed that, compared to the control group, patients with MDD exhibited increased NH in the overlapping regions of the MPFC and anterior DMN (Guo et al., 2018). In individuals with treatment‐resistant MDD, functional studies consistently reveal elevated activity within the MPFC (Phillips et al., 2015). Furthermore, heightened FC within the anterior DMN (including the left superior MPFC) persisted in MDD patients even after 12 weeks of antidepressant treatment (Li et al., 2013). This heightened activity can be reversed through stimulation of the subcallosal cingulate cortex, leading to the amelioration of depressive symptoms (Hadas et al., 2019; Morris et al., 2020). The primary explanation for this phenomenon is rooted in the MPFC integration of inputs from various brain regions as well as its projection of neurons to both cortical and limbic structures. Via these intricate connections, the MPFC governs an array of cognitive and behavioral functions, including attention, habit formation, decision‐making, memory, processing of aversive and appetitive stimuli, and the regulation of emotions. Alterations in these processes are linked to behaviors reminiscent of depression (Bittar & Labonte, 2021). Consequently, our hypothesis posits that the increased NH activity in the left superior MPFC could impact the operational dynamics of this region, potentially causing a disturbance in the coordination between the cognitive and emotional systems. Our earlier research outcomes have further supported and validated this hypothesis (Fu et al., 2021; Guo et al., 2018).

The PCC assumes a crucial role within the DMN, displaying strong connectivity with the entorhinal cortex and parahippocampal gyrus in primates. This connectivity exerts influence on the hippocampal memory system (Bubb et al., 2017; Vogt & Pandya, 1987). Primarily associated with autobiographical memory, the PCC also plays a key part in sustaining self‐awareness. Moreover, it guides introspective thought during periods of rest and contributes to the regulation of diverse aspects, including cognition, emotion, action, and intuition (Leech & Sharp, 2014). Previous studies have reported elevated rfFC within the DMN in patients with MDD (Nejad et al., 2013; Sheline et al., 2010; Zhou, Yu et al., 2010). The hyperactivity of the DMN is linked with rumination in patients diagnosed with MDD (Broyd et al., 2009). In individuals with MDD, rumination and negative emotions mutually influence and amplify each other, particularly in impulsive individuals, ultimately leading to impulsive behaviors (Selby et al., 2016). Li, Zhang et al. (2021) illustrated that, in contrast to HCs, individuals with MDD displayed heightened FC in the bilateral PCC. Furthermore, studies have shown that elevated ReHo values in the PCC/precuneus and middle cingulate cortex within the DMN signify a compensatory reaction to emotional regulation and self‐perceptions in individuals experiencing MDD along with sleep disturbances. This compensatory response may contribute to challenges in initiating sleep and experiencing suboptimal sleep quality (Wang et al., 2020). Therefore, we posit that the elevated NH in the PCC may impact on the functionality of this region, representing a characteristic feature in individuals with MDD.

In our investigation, we noted noteworthy occurrences: Clinical variables demonstrated a positive correlation with FC in the brains of individuals with depression (though significance diminished following the Bonferroni correction), notably blood lipid levels. Consistent with our previous research, we propose that lipids present in peripheral blood could influence brain function through dual pathways. Initially, they may compromise the integrity of the blood–brain barrier, instigating inflammation and neurotoxic reactions within the central nervous system. Subsequently, they could induce overactivation of the hypothalamus–pituitary–adrenal axis, resulting in heightened hormone levels in the central nervous system and the attenuation of negative feedback regulation. This, in turn, may lead to metabolic issues such as elevated blood sugar (Li, Teng et al., 2021). It is plausible that, among patients with MDD, the impact of depressive symptoms may contribute to a diminished appetite and reduced food intake, culminating in decreased lipid levels.

Prior research suggests that in the HCs group, no evident link exists between dynamic connectivity states and personality. In contrast, a distinct correlation emerges in individuals diagnosed with MDD (Wu et al., 2019). Our study aligns with and reinforces this observation. Notably, personality traits may be interconnected with the SN of the brain (Tian et al., 2018). The salience network assumes a pivotal role in the individual's shift from external information processing in the “conscious” to “awareness” states, serving as a pivotal switch between the self‐monitoring network and task‐related networks (Palaniyappan & Liddle, 2012). Researchers have extensively employed positron emission tomography to delve into the positive correlation between distinct brain regions and personality traits (Johnson et al., 1999). This implies that modifications in brain network connections might result in changes to personality traits.

Individuals experiencing MDD may exhibit social skill deficiencies characterized by delayed speech in dialogue, restricted eye contact, and challenges in focusing on conversation topics. These manifestations are attributed to an elevated self‐focus (Mor & Winquist, 2002). Depressed individuals might face limitations in cognitive resources, hindering their ability to process external signals. The struggle to disengage from internal thoughts and emotions can lead to individuals with self‐focused depressive symptoms appearing bothersome and abrasive to friends, thereby complicating interpersonal relationships (Schwartz‐Mette & Rose, 2016). In contrast, individuals with MDD demonstrate deactivation of μ‐opioid receptors (MOR) in the amygdala, a phenomenon potentially associated with heightened reactivity to negative social cues, such as peer rejection, according to research findings (Hsu et al., 2013). Additionally, when compared to HCs, MDD patients display MOR deactivation in the amygdala, which could be linked to heightened blood‐oxygen‐level‐dependent hyperactivity in response to negative social cues like peer rejection (Hsu et al., 2015). Additional investigations have showcased functional abnormalities in diverse brain regions, such as the subgenual anterior cingulate cortex (Masten et al., 2011), insula (Silk et al., 2014), dorsolateral prefrontal cortex (Hooley et al., 2005), and striatum (Gradin et al., 2015). These abnormalities have repercussions on the social functioning of individuals diagnosed with MDD. Our research findings indicate a negative correlation between the left PCC and scores of utilization of social support. This offers a novel direction for investigating abnormal brain connectivity and social functioning.

In summary, despite a lack of direct evidence, the personality and lipid level in patients with MDD may still be associated with functional changes in the DMN, as indicated by this study. This finding could potentially offer some evidence for distinguishing between MDD and HCs in the future.

This study has several limitations. First, a relatively small sample size was employed at the same time, we did not divide the MDD sample into first‐episode and recurrent patients. Second, due to issues with patient selection for enrollment, it is difficult to effectively control the age differences among participants, which may potentially impact the research results. Therefore, future studies need to rigorously define the age criteria for enrolling participants. Third, the research focused exclusively on DMN, potentially overlooking changes in FC in other brain regions. Lastly, this is a cross‐sectional study, limiting the exploration of the relationship between abnormal short‐term and long‐term NH, disease progression, and the impact of treatment on MDD.

## 5 CONCLUSION

The study results indicate abnormalities in NH within the DMN in individuals with MDD. These abnormalities could potentially serve as candidate biomarkers to distinguish patients from HC participants. Therefore, the findings underscore the significance of the DMN in the pathophysiology of MDD. Additionally, our research explores the correlation between clinical variables and FC in the brains of depressed patients, providing preliminary evidence and suggesting a new direction for future investigations.

## AUTHOR CONTRIBUTIONS

**Chunguo Zhang**: Writing—review and editing; writing—original draft; methodology; software. **Feichao Ruan**: Methodology; software; writing—original draft; writing—review and editing. **Haohao Yan**: Software; writing—review and editing; writing—original draft; methodology. **Jiaquan Liang**: Validation; investigation; resources. **Xiaoling Li**: Investigation; validation; resources. **Wenting Liang**: Investigation; validation; resources. **Yangpan Ou**: Investigation; validation; resources. **Caixia Xu**: Investigation; validation; resources. **Guojun Xie**: Supervision; project administration; funding acquisition. **Wenbin Guo**: Supervision; project administration; funding acquisition.

## CONFLICT OF INTEREST STATEMENT

The authors declare that the research was conducted in the absence of any commercial or financial relationships that could be construed as a potential conflicts of interest.

### PEER REVIEW

The peer review history for this article is available at https://publons.com/publon/10.1002/brb3.3622.

## Supporting information

Supporting Information

## Data Availability

The datasets used and/or analyzed during this study are available from the corresponding author (Guo W) upon reasonable request.
