# Supplementary material for: Potential correlations between abnormal homogeneity of default mode network and personality or lipid level in major depressive disorder
Source: Brain Behav. 2024 Jul 17;14(7):e3622. doi: 10.1002/brb3.3622 (PMC11255032; doi:10.1002/brb3.3622)
Supplement: Supplementary file 1 — Supporting Information [file BRB3-14-e3622-s001.docx]

Potential correlations between abnormal homogeneity of default mode network and personality or lipid level in major depressive disorder

Chunguo Zhang^1^ †, Feichao Ruan^1^ †, Haohao Yan^2^ †, Jiaquan Liang^1^, Xiaoling Li^1^, Wenting Liang^1^, Yangpan Ou^2^, Caixia Xu^1^, Guojun Xie^1^*, Wenbin Guo^2^*

**Affiliation/address:**

^1^Department of Psychiatry, The Third People's Hospital of Foshan, Foshan, Guangdong 528000, China.

^2^ Department of Psychiatry, National Clinical Research Center for Mental Disorders, and National Center for Mental Disorders, The Second Xiangya Hospital of Central South University, Changsha 410011, Hunan, China.

†: Chunguo Zhang, Feichao Ruan, and Haohao Yan contributed equally to this work.

***Correspondence:**

Guojun Xie

Department of Psychiatry, The Third People's Hospital of Foshan, Foshan, Guangdong 528000, China.

Email: xiegjfs@126.com

Wenbin Guo

Department of Psychiatry, National Clinical Research Center for Mental Disorders, and National Center for Mental Disorders, The Second Xiangya Hospital of Central South University, Changsha 410011, Hunan, China.

Email: guowenbin76@csu.edu.cn


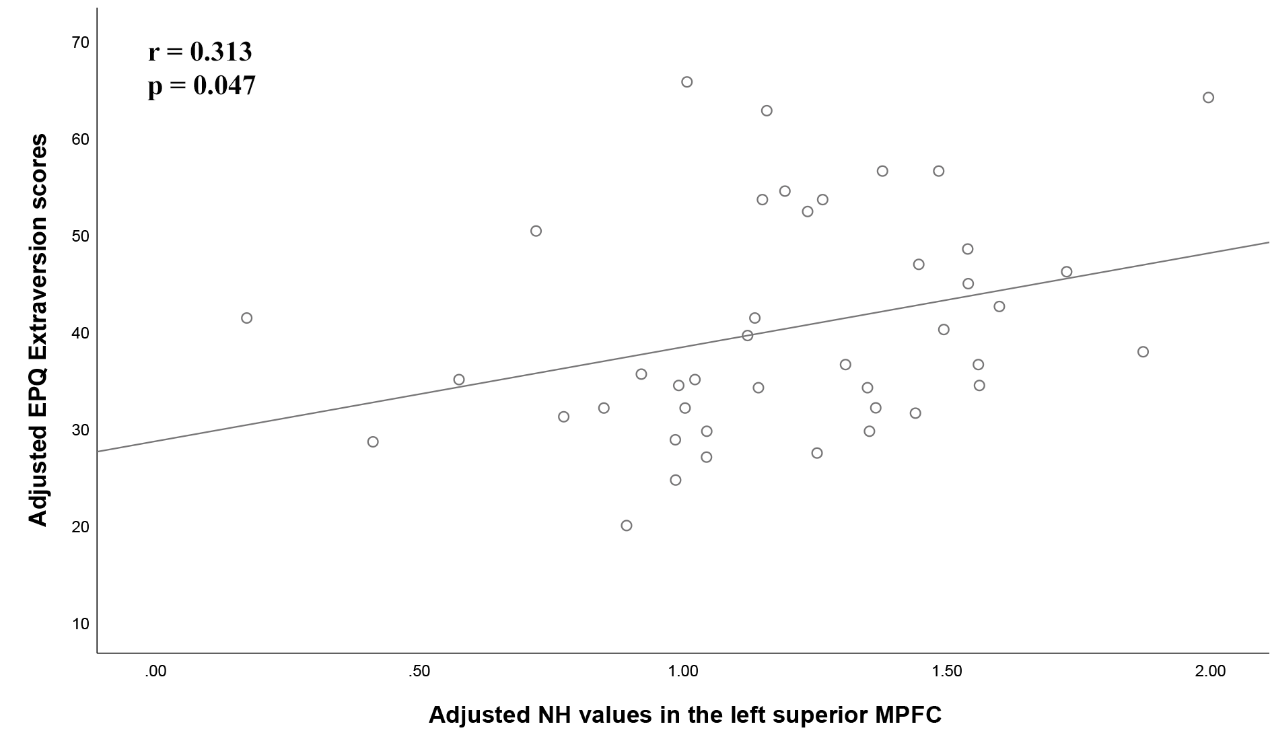


Figure S1: Partial correlation analysis between the network homogeneity values of the left superior Medial Prefrontal Cortex and EPQ Extraversion scores, controlling for age.


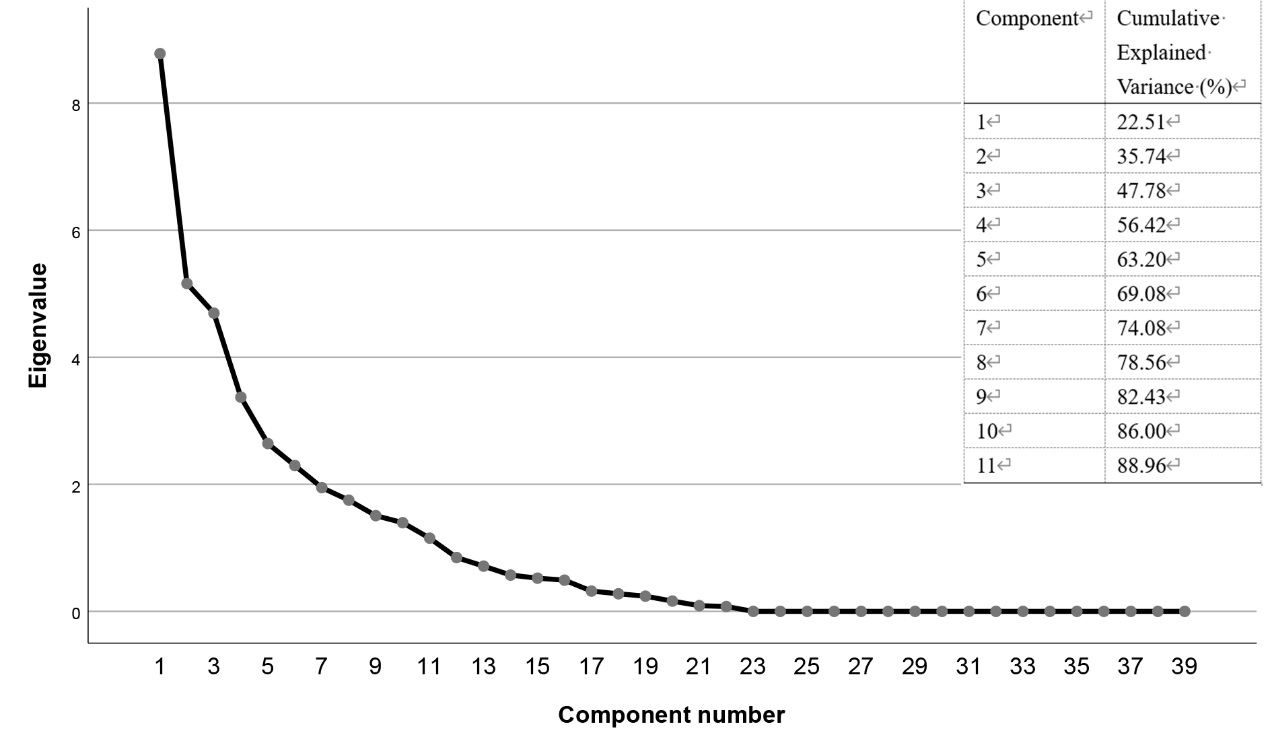


Figure S2: Principal component analysis and the cumulative explained variance.

Table S1. Correlation analysis between abnormal network homogeneity values and the factors derived from the principal component analysis of the clinical variables.

| Factors | Left superior medial prefrontal cortex (r/rho, p) | Left posterior cingulate cortex (r/rho, p) |
| --- | --- | --- |
| Factor 1 | -0.151, 0.340^a^ | -0.293, 0.060^a^ |
| Factor 2 | 0.185, 0.241^a^ | -0.175, 0.266^a^ |
| Factor 3 | -0.044, 0.780^a^ | 0.012, 0.941^a^ |
| Factor 4 | 0.254, 0.104^b^ | -0.085, 0.591^b^ |
| Factor 5 | -0.014, 0.930^a^ | -0.141, 0.374^a^ |
| Factor 6 | 0.144, 0.364^a^ | 0.005, 0.973^a^ |
| Factor 7 | -0.020, 0.898^a^ | 0.003, 0.984^a^ |
| Factor 8 | -0.161, 0.308^b^ | -0.115, 0.467^b^ |
| Factor 9 | -0.175, 0.267^a^ | -0.068, 0.669^a^ |
| Factor 10 | 0.243, 0.121^a^ | -0.183, 0.246^a^ |
| Factor 11 | -0.153, 0.334^a^ | -0.098, 0.537^a^ |

^a^ Pearson correlation.

^b^ Spearman correlation.

Table S2. Partial correlation analysis between abnormal network homogeneity values and the factors derived from the principal component analysis of the clinical variables, controlling for age.

| Factors | Left superior medial prefrontal cortex (r, p) | Left posterior cingulate cortex (r, p) |
| --- | --- | --- |
| Factor 1 | -0.109, 0.497 | -0.264, 0.096 |
| Factor 2 | 0.261, 0.099 | -0.131, 0.414 |
| Factor 3 | -0.123, 0.442 | -0.051, 0.753 |
| Factor 4 | 0.171, 0.284 | -0.085, 0.597 |
| Factor 5 | -0.011, 0.948 | -0.141, 0.378 |
| Factor 6 | 0.072, 0.656 | -0.064, 0.689 |
| Factor 7 | 0.083, 0.605 | 0.092, 0.568 |
| Factor 8 | -0.092, 0.568 | -0.155, 0.333 |
| Factor 9 | -0.087, 0.588 | 0.016, 0.923 |
| Factor 10 | 0.290, 0.059 | -0.136, 0.397 |
| Factor 11 | -0.063, 0.695 | -0.018, 0.909 |
